# A Case Study of a Hepatitis B Screening and Blood Pressure Assessment Program in Los Angeles County, 2012–2013

**DOI:** 10.5888/pcd12.140373

**Published:** 2015-02-12

**Authors:** Noel C. Barragan, Mimi Chang, Jennifer Felderman, Heather Readhead, Tony Kuo

**Affiliations:** Author Affiliations: Mimi Chang, Asian Pacific Liver Center at St. Vincent Medical Center, Los Angeles, California; Jennifer Felderman, Division of HIV and STD Programs, Los Angeles County Department of Public Health, Los Angeles, California; Heather Readhead, The Wellness Center at the Historic General Hospital, Los Angeles, California; Tony Kuo, Los Angeles County Department of Public Health, Los Angeles, California, David Geffen School of Medicine at UCLA, Los Angeles, California, and UCLA Fielding School of Public Health, Los Angeles, California.

## Abstract

The Los Angeles County Department of Public Health teamed with a culturally tailored, community-based organization to augment their hepatitis B screening program with blood pressure assessments. During 6 months, 2,298 people were served by the program; descriptive statistics and models were generated to describe demographics and screening and assessment results. Despite the program having good reach, sustainability was challenging. This experience draws attention to the need for invested desire to change at both the organizational and patient levels to sustain interdisciplinary provision of clinical preventive services.

## Objective

The Centers for Disease Control and Prevention’s 2012 Community Transformation Grants (CTG) program sought to improve health by addressing 3 strategic directions, including the ABCs of prevention (aspirin use, blood pressure control, cholesterol management, and smoking cessation) (1).

As a CTG grantee, the Los Angeles County Department of Public Health supported strategies aimed at enhancing clinical preventive services. One such effort included extending services to hard-to-reach Asians by partnering with a culturally tailored, community-based organization to include blood pressure assessments in their long-standing hepatitis B screening program. This report describes this program and lessons learned.

## Methods

Augmentation of the existing program began in August 2012. In the ensuing 6 months, 2,298 people were offered screening for hepatitis B surface antigen (HBsAg) and hepatitis B surface antibodies (anti-HBs) at 26 community health events; at 18 events, 1,499 people were also offered blood pressure assessments. Blood pressure assessments were not offered at 8 events because other organizations were providing this service. Hepatitis testing was administered by certified and licensed staff, and blood pressure measurements were taken by trained volunteers using an automated sphygmomanometer. Events were held in primarily Asian communities, but services were available to any person regardless of race/ethnicity. People interested in screening or assessment were given educational information and asked to complete a self-administered intake questionnaire, which included questions about date of birth, sex, race/ethnicity, and marital status, in the appropriate language.

Hepatitis B results were mailed directly to participants and/or their referring physician within 2 or 3 weeks of screening. Blood pressure results were available immediately. Those who screened positive for hepatitis B or possible hypertension were referred to local clinics for follow-up, using standardized clinical protocols. Protocols for the case study were reviewed and approved by the Los Angeles County Department of Public Health institutional review board.

Descriptive statistics were generated to describe demographics and screening and assessment results. Contingency tables of HBsAg and anti-HBs results were created to stratify disease staging (2). A multivariable logistic regression model, which included data on 2,013 participants, was constructed to describe factors associated with a positive HBsAg test. A generalized ordered logit analysis was performed to examine factors that influenced blood pressure results; of those who received assessments, 1,178 had complete data and were included in the model. Because the proportional odds assumption was not met for all variables in the ordered model, the less restrictive, user-written “gologit2” program was used (3). All data analyses were conducted using Stata, version 12.0 (StataCorp LP).

## Results

Most program participants (61.2%, n = 1,407) were women. More than half (n = 1,385) were aged 45 to 64 years, and most were married (62.5%, n = 1,437) (Table 1).

**Table 1:** Characteristics of Participants in a Hepatitis B Screening and Blood Pressure Assessment Program in Los Angeles County, 2012–2013^a^

Demographics and Screening and Assessment Results	All Participants (N = 2,298)	Participants Screened for Hepatitis B (n = 2,232)	Participants Assessed for High Blood Pressure (n = 1,318)
**Age, y**			
<18	23 (1.0)	22 (1.0)	0
18–44	444 (19.3)	430 (19.3)	240 (18.2)
45–64	1,385 (60.3)	1,344 (60.2)	787 (59.7)
65–74	327 (14.2)	320 (14.3)	215 (16.3)
75–84	106 (4.6)	103 (4.6)	70 (5.3)
≥85	13 (0.6)	13 (0.6)	6 (0.5)
**Sex**			
Female	1,407 (61.2)	1,357 (60.8)	811 (61.5)
Male	889 (38.7)	874 (39.2)	507 (38.5)
**Race/ethnicity** b			
Southeast Asian	1,034 (45.0)	1,004 (45.0)	742 (56.3)
Chinese/Taiwanese	737 (32.1)	726 (32.5)	370 (28.1)
Other Asian	255 (11.1)	246 (11.0)	34 (2.6)
Other	162 (7.0)	149 (6.7)	99 (7.5)
**Marital status**			
Single or widowed	685 (29.8)	660 (29.6)	419 (31.8)
Married	1,437 (62.5)	1,402 (62.8)	792 (60.1)
**Hepatitis B results**			
**HBsAg**			
Positive	—	159 (7.1)	—
Negative	—	2,073 (92.9)	—
**Anti-HBs**			
Positive	—	1,458 (65.3)	—
Negative	—	774 (34.7)	—
**Blood pressure readings** c			
Normal	—	—	483 (36.7)
Prehypertension	—	—	456 (34.6)
Stage 1 hypertension	—	—	284 (21.6)
Stage 2 hypertension	—	—	95 (7.2)

Abbreviations: —, not applicable; HBsAg, hepatitis B surface antigen; anti-HBs, hepatitis B surface antibodies.

a All values are numbers and percentages; they may not add to 100% because of missing values or rounding.

b Southeast Asian includes Burmese, Cambodian, Filipino, Laotian, Thai, and Vietnamese; “other Asian” includes Armenian, Bangladeshi, Indian, Japanese, Korean, Lebanese, and Mongolian; “other” includes African American, white, Hispanic, Native American, Ukrainian, and multiracial people.

c Hypertension classification as defined by the Seventh Report of the Joint National Committee on Prevention, Detection, Evaluation, and Treatment of High Blood Pressure (4).

Of those screened (n = 2,232), 159 participants tested positive for HBsAg; 1,458 tested positive for anti-HBs (Table 1); 1,445 participants were immune and 628 (who had no immunity) were eligible for vaccination. Tests suggested that 146 participants had an active chronic infection that would possibly require life-long follow-up and treatment (5).

Of the 1,499 participants who were offered blood pressure assessments, 1,318 had their measurements taken. Of these, 34.6% had readings in the prehypertension range; 28.8% had readings in the hypertension range (Table 1). Forty-two of these latter cases were also HBsAg positive.

In the regression analysis of HBsAg results (n = 2,013), race/ethnicity was associated with a positive test (Table 2). When stratified by race/ethnicity, “other Asian” and “other” groups were less likely than Southeast Asians to test positive for HBsAg (*P <* .05). Those aged 65 or older also were less likely than those aged 44 or younger to test positive (adjusted odds ratio [AOR] = 0.52; 95% confidence interval [CI], 0.28–0.96; *P* = .03). Men were more likely than women to test positive (AOR = 1.67; 95% CI, 1.19–2.36; *P* = .003).

**Table 2:** Factors Associated With Positive Screening Results Among Participants in a Hepatitis B Screening and Blood Pressure Assessment Program, Los Angeles County, 2012–2013

Independent variable	HBsAg Positive, AOR (95% CI) (N = 2,013)^a^	Normal vs Prehypertension and Hypertension^b^ Coefficient (95% CI) (n = 1,178)^c^	Normal and Prehypertension vs Hypertension^b^ Coefficient (95% CI) (n = 1,178)^c^
**Race/ethnicity^d^**			
Chinese/Taiwanese	0.90 (0.63 to 1.30)	−0.11 (−0.36 to 0.14)	−0.11 (−0.36 to 0.14)
Other Asian	0.32^e^ (0.15 to 0.71)	0.48 (−0.20 to 1.16)	0.48 (−0.20 to 1.16)
Other	0.23^f^ (0.07 to 0.74)	−0.21 (−0.63 to 0.20)	−0.21 (−0.63 to 0.20)
Southeast Asian	1.0 [Reference]	1.0 [Reference]	1.0 [Reference]
**Age, y**			
45–64	1.04 (0.67 to 1.60)	0.90^g^ (0.60 to 1.21)	0.90^g^ (0.60 to 1.21)
≥65	0.52^h^ (0.28 to 0.96)	1.83^g^ (1.40 to 2.25)	1.07^g^ (0.67 to 1.47)
≤44	1.0 [Reference]	1.0 [Reference]	1.0 [Reference]
**Sex**			
Male	1.67^i^ (1.19 to 2.36)	0.76^g^ (0.49 to 1.02)	0.44^g^ (0.18 to 0.70)
Female	1.0 [Reference]	1.0 [Reference]	1.0 [Reference]
**Marital status**			
Married	1.16 (0.79 to 1.70)	−0.10 (−0.34 to 0.13)	−0.10 (−0.34 to 0.13)
Single or widowed	1.0 [Reference]	1.0 [Reference]	1.0 [Reference]

Abbreviations: AOR, adjusted odds ratio; CI, confidence interval; HBsAg, hepatitis B surface antigen.

a Logistic regression model.

b Hypertension classification as defined by the Seventh Report of the Joint National Committee on Prevention, Detection, Evaluation, and Treatment of High Blood Pressure (4).

c Generalized ordered logit model.

d Southeast Asian includes Burmese, Cambodian, Filipino, Laotian, Thai, and Vietnamese; “other Asian” includes Armenian, Bangladeshi, Indian, Japanese, Korean, Lebanese, and Mongolian; “other” includes African American, white, Hispanic, Native American, Ukrainian, and multiracial people.

e *P* = .005.

f *P* = .01.

g *P* ≤ .001.

h *P* = .03.

i *P* = .003.

No significant association was found between race/ethnicity or marriage status and blood pressure readings. However, men and participants aged 65 or older (compared with those aged ≤44) were more likely to have blood pressure readings in the prehypertension and hypertension ranges (*P* ≤ .001) (Table 2).

## Discussion

The integration of blood pressure assessments to an existing hepatitis B screening program reached a good number of high-risk people during the 6-month study period. About 7% of those screened for hepatitis B were thought to have active chronic infection; however, disease severity, including the degree of portal hypertension and liver cirrhosis, was not assessed in this study. Another 28% had no immunity and could benefit from vaccination. Almost two-thirds of those assessed for blood pressure had readings in the prehypertension or hypertension ranges. A subgroup analysis suggested that treatment was not uniform. For example, of 42 Chinese adults with readings in the hypertension range, only 22 reported taking blood pressure medications. These and other results from the models were consistent with the literature, further highlighting the risks associated with these conditions (4,6,7).

Building on a well-established community-based effort enabled the Los Angeles County Department of Health to engage a high-risk segment of the Asian community that has limited access to and limited use of high-quality medical care because of socioeconomic constraints, language barriers, and cultural beliefs (8). Despite the benefits of this augmented program, sustainability was a challenge. Program infrastructure made field implementation of blood pressure screening relatively simple and efficient; however, challenges arose when participants were reluctant to lengthen their encounter time to receive the additional services. To maintain client satisfaction and participation in the hepatitis B screening, the blood pressure assessments were discontinued. This experience draws attention to the need for an invested desire for change at both the organizational and patient levels. A shift in this social norm is essential for sustaining interdisciplinary provision of clinical preventive services.

This enhanced program underscores the value of bringing together multiple partners (eg, health care, public health, the community) to leverage resources and develop community–clinical linkages. For this model of practice to be sustainable, coordination of investments, services, and innovation in the community should be strengthened. Furthermore, a shift in the collective attitude toward supporting these and other prevention efforts is needed.
